# Fracture of the Head of Humerus, Exsection, Etc.

**Published:** 1890-12

**Authors:** A. N. Denton

**Affiliations:** Austin


DANIEL'S

### Texas Medical Journal.

A Representative Organ of the Medical Profession, and an Exponent of Rational Medicine; devoted to the Organization, Advancement and Elevation of the Profession in Texas.

PUBLISHED MONTHLY.—SUBSCRIPTION $2.00 A YEAR.

Vol. VI. AUSTIN, DECEMBER, 1890. No. 6.

### Original Articles.

CONTRIBUTED EXCLUSIVELY TO THIS JOURNAL.

*The Articles in this Department are accepted and published with the understanding that we are not responsible for, nor do we indorse the views and opinions of the writers by so doing.*

For Daniel's Texas Medical Journal.

### FRACTURE OF THE HEAD OF THE HUMERUS, ETC.

BY A. N. DENTON, M. D., AUSTIN.

Read before the Austin District Medical Society, June 19, 1890.

ABOUT January 25, 1890, Mr. A. M. A., while riding on the prairie near Dripping Springs, Hays county, threw a lasso over the horns of a three years old steer, was dragged from his horse, and fell heavily among the mass of comb-rocks which abound in that locality.

The violence of the shock rendered him completely unconscious for about thirty or forty minutes. His companion placed him in a wagon and conveyed him to his ranch, a mile or two distant. After the return of consciousness, several hours elapsed before the restoration of any memory of the accident or its cause. Serious injury was not suspected by the friends of the young man, and surgical assistance was not deemed necessary.

Pain in the shoulder-joint, fever, loss of appetite, however, rapidly supervened, and on January 31, I was requested by the mother of the young man to visit him at his ranch.

I found him on the morning of February 1st with a temperature of 103°, pulse 120. Shoulder quite tender and greatly swollen, no appetite and bowels constipated. After as careful and complete an examination as was practicable with the inflamed and swollen condition of the shoulder, I decided that a dislocation of the humerus downward existed, and advised his immediate removal to Austin, which was done on February 1.

On February 2nd Dr. T. D. Wooten was invited to examine the patient, which he did with his usual care, and coincided with me in the diagnosis.

During the intense inflammation and great swelling it was thought best to postpone any attempt at reduction, until measures should be employed to reduce the swelling and abate the fever. Accordingly general and local treatment to this end was directed and faithfully carried out. Under the use of these measures the feversome what abated, and the swelling of the arm was greatly reduced. The inflammation became more local and the heat and pain in the shoulder more intense. The pain became so constant and excruciating as to require the daily use of morphine in order to procure rest.

On about the sixth day after the arrival of the patient in Austin, a second examination was made by Dr. Wooten and myself, and the existence of pus in and about the joint being suspected, a hypodermic needle was introduced, and pus withdrawn into the barrel of the syringe. After consultation it was thought best to evacuate the pus by a free incision, which was made in front of, and below the coracoid process. About eight ounces of pus was evacuated and a drainage tube was inserted and left in the wound. Two days later a large abscess having appeared on the posterior aspect of the shoulder, another incision was made, and six or eight ounces of pus escaped.

The patient was now greatly prostrated and fever of rather a high grade continued. About the 12th of February it was decided to attempt to reduce the supposed dislocation, which was accomplished under the influence of chloroform.

No amendment of the symptoms, however, followed the reduction. Profuse suppuration continued, notwithstanding the joint was thoroughly sponged twice daily with a solution of boracic and carbolic acid, the detergent fluid passing in at one orifice and out at the other, apparently traversing the joint and intermediate structures. Intense pain continued, and emaciation and loss of strength progressed, in the face of the most generous diet and supporting treatment.

For nearly two months the patient was bedridden and consumed by hectic fevers. Bed sores supervened and the case seemed to be moving slowly on toward a fatal termination. However, about the end of the second month from the date of the accident his appetite began to improve and he was induced to sit up in a chair for a short time each day, at first for only a few moments, but the time was gradually lengthened until his strength so increased as to enable him to walk about the yard and to keep off the bed for the greater part of each day. Meantime morphine was administered hypodermically every night, and his bowels were kept open by injections of warm water.

During the last four weeks prior to the operation he gained a little in strength and flesh, but there was little or no diminution in the suppuration, and drainage tubes were kept in the fistulary openings leading down to the bone. The syringing was also kept up night and morning; by this means the wound was kept moderately aseptic, and the absorption of pus was in a great measure prevented.

About the 18th of April the patient had a sharp attack of dysentery, which greatly reduced his slender store of vitality. For several weeks I had been convinced that necrosis of the bone existed, and was only waiting for the most favorable opportunity to operate.

After partial recovery from the above mentioned attack of dysentery, believing that nothing could be gained from a further postponement, I determined to excise the joint. Accordingly on the evening of April 23d, assisted by Drs. Litten, Gazley and

Bennett, of this city, after first demonstrating the existence of necrosed bone by the use of the probe, I proceeded to operate.

Of the three methods mentioned by authors of excising this joint, I selected the most difficult of execution, because it involved less injury to the muscles and ligaments about the joint.

The incision was begun on the lower extremity of the accromion process, and carried down the arm through the center of the deltoid to its insertion (about five inches), and down to the bone, carefully dissecting through the deltoid, so as to avoid a division of its fibres, and thus injuring its future integrity and usefulness. Having divided the soft parts down to the joint the circumflex was felt for, but not found, having been disintegrated, and destroyed by the long continued suppuration. The long head of biceps was also destroyed by the suppurative process.

In dividing the muscular tissues near the bone below the joint, the posterior circumflex artery was divided, and troublesome hemorrhage was for a time encountered owing to the depth and narrowness of the wound.

It was however, at length taken up and tied. With the left forefinger hooked over the end of the bone in the joint, and with the right hand grasping the elbow, the end of the bone was forced through the wound, and was found to be denuded of periosteum for a distance of nearly three inches from its upper extremity, and was divided with the saw about four inches below the joint. At this stage of the operation, having thrust the left forefinger into the joint, a rough surface was felt, and for a moment disease and necrosis of the acetabulum was suspected, but by further manipulation the finger was hooked over the remnants of the head of the bone, and it was withdrawn from its bed, and is here exhibited. After the extraction of this fragment, the acetabulum was found to be smooth, and uninjured.

A portion of the corracoid process, however, was found to be diseased, and was removed. The cavity was well syringed out with a solution of boracic and carbolic acid, and the external wound was closed with nine silk sutures.

A drainage tube was left in the wound, the upper end of which was at the former joint, and the other was made to pass out at the lower angle of the wound.

The patient was profoundly prostrated, and his remaining vital powers was severely taxed by the shock of the operation, but under the use of appropriate remedies reaction was slowly established, and on the following morning his condition was satisfactory.

The progress toward recovery was rapid and uninterrupted. The entire tract of the incision, down to the drainage tube adhered and healed by first intention. The large cavity from which the bone was taken, rapidly filled up, and at the end of two weeks from the operation suppuration had entirely ceased.

The improvement in the general health of the patient was phenomenal. At the end of the third week he was well and returned to his business at his ranch in the mountains. I have since learned that he is rapidly regaining the use of the arm.

This case is presented to the society, not on account of its rarity, as exsection of the shoulder joint is common enough both in military and civil practice. But aside from this, the case presents some interesting and peculiar features. The failure to form a correct diagnosis is especially noteworthy. I confess that after a careful examination, I failed to detect a fracture of the head of the bone, which evidently existed, and even after repeated examinations, fracture was scarcely suspected; and this failure to detect an important pathological condition is the more surprising, as the patient repeatedly passed under the hand and observation of Dr. T. D. Wooten, of this city, whose great experience and painstaking care renders him less liable to mistakes than the writer.

It has been suggested that possibly there was no fracture, but that the head of the bone may have separated from the shaft at the epiphesis from the disintegrating effect of long continued suppuration. But this hypothesis is, in my judgment, extremely improbable, to say the least.

It is far more rational to conclude that the fracture occurred at the time of the accident, and that the head of the bone remaining in the acetabulum without vascular connection acted as a foreign body, and was the cause of the suppuration. When the lower fragment was reduced and placed in opposition to the head of the bone, of course no union could take place, but destructive disintegration and suppuration continued. This destructive process was promoted and hastened by the upward pressure of the lower fragment against the head of the bone, until it became a mere shell, as you see. The effect of this continued pressure is also apparent on the upper end of the lower fragment as you may observe. The failure to diagnose the true nature of the injury in this case was due to lapse of time between the injury and the first examination, and the consequent great swelling and inflammation, rendering it difficult to manipulate the joint. Some comfort may be derived from the fact that the final termination of the case was not disastrous, or materially prejudiced by the mistake.

There is no denying the fact, however, that if the true condition of the joint had been ascertained at an earlier date, the operation should and would have been performed much earlier, and the patient might have been saved much needless suffering, and probably a more useful arm would have resulted.

